# Effects of Epidural Analgesia on Labor Pain and Course of Labor in Primigravid Parturients: A Prospective Non-randomized Comparative Study

**DOI:** 10.7759/cureus.26090

**Published:** 2022-06-19

**Authors:** Dipika Deepak, Archana Kumari, Rajat Mohanty, Jay Prakash, Tushar Kumar, Shio Priye

**Affiliations:** 1 Obstetrics and Gynecology, Rajendra Institute of Medical Sciences, Ranchi, IND; 2 Obstetrics and Gynecology, Blue Wheel Hospital, Bhubaneshwar, IND; 3 Critical Care Medicine, Rajendra Institute of Medical Sciences, Ranchi, IND; 4 Anesthesiology, Rajendra Institute of Medical Sciences, Ranchi, IND

**Keywords:** comparision, cousre of labor, primigravid parturients, labor pain, epidural analgesia

## Abstract

Background and aim: The aim of this study was to compare the effects of epidural analgesia on relief of labor pain, progress, and outcome of labor in primigravid parturients to those who did not receive any analgesia.

Methods: This was a hospital-based, quasi-experimental study conducted on 70 primigravid parturients at term with a single fetus in a cephalic presentation in active labor. Parturients who were willing to receive epidural analgesia formed group S (n=35) and parturients who refused epidural analgesia formed group C (n=35). The primary objective was to compare alleviation of pain measured by the Visual Analogue Scale (VAS) score. Secondary objectives were to compare the duration of labor, mode of delivery, and neonatal outcome.

Results: Pain intensity was significantly lower in group S compared to group C at all measured points of time (p<0.001). There was a quick fall in mean VAS score in group S from 7.94 to 3.86 within 20 min with the bolus dose, it further dropped to 1.03 after 3 h. Further, 88.6% of parturients in groups rated their pain relief as excellent and good satisfaction score. Prolongation of active phase of the first stage of labor (>6 h) was not significant (17.1 % in epidural group versus 5.7% in control group; p=0.259). However, prolongation of the second stage of labor (> 2h) was significant (18.2% in study group versus 0% in control group; p=0.024). The rate of cesarean section, instrumental vaginal delivery, and neonatal outcome was similar in both groups. No adverse effects were observed on maternal vitals, fetal heart rate and Apgar score at 5 min.

Conclusion: Epidural analgesia alleviated labor pain in all primigravid parturients who opted for it, without an increase in cesarean section and instrumental vaginal birth. Improved parturients' satisfaction with associated neonatal safety provides a positive birth experience. There was no effect on duration of active phase of the first stage of labor, but the duration of the second stage of labor was slightly prolonged.

## Introduction

Labor pain is undoubtedly the most severe and worst kind of pain that most women will ever experience in their lives. Hence, alleviating labor pain is one of the key concerns for parturients and their family members. The need to provide effective and safe labor analgesia continues to be a challenge for both obstetricians and anesthesiologists. Effective pain relief during labor improves the woman's satisfaction with a positive birth experience.

Numerous pharmacologic and non-pharmacologic methods (transcutaneous electrical nerve stimulation {TENS}, massage, acupuncture, water immersion, water birth, yoga, music therapy, biofeedback, continuous labor support by midwives, positioning, ambulation, hypnosis, and aromatherapy) have been used for relief of labor pain [1]. Labor analgesia has traveled a long path from older days of ether and chloroform in 1847 to the present-day labor pain management using evidence-based medicine. During labor, epidural analgesia provides effective pain relief [2-4]. The most widely used method of pain relief in labor is lumbar epidural analgesia [5]. However, the use of epidural analgesia is surrounded by controversies like delay in labor progress, increased rate of operative intervention and instrumental delivery, and harmful effects on fetus and newborn. Epidural analgesia does have some side effects like headache, soreness in back, itchiness, backache, leg numbness, transient urination problems, and a decrease in blood pressure. In extremely rare situations, they may result in complications such as permanent nerve damage. These factors affect the use of epidural analgesia for painless labor.

In developed countries, epidural analgesia is popular and widely used for pain relief in parturients [6,7]. According to the American College of Obstetricians and Gynecologists, maternal request can be taken as sufficient indication for pain relief during labor in the absence of medical contraindications [8]. In developing countries like India, the awareness or acceptance of pain-relieving options for parturients is very sparse in public health facilities, and only few private centers have a labor analgesia program. With this background, the present study was designed with the aim to compare the effects of epidural analgesia on labor pain relief, progress, and outcome of labor in primigravid parturients compared to those not receiving any analgesia.

## Materials and methods

This study was a prospective non-randomized comparative study conducted from March 2018 to August 2019 after approval from Institutional Ethics Committee (approval number: IEC70, dated: February 20, 2018). Methods of epidural analgesia were formulated in collaboration with the Department of Anesthesia. During the study period, primigravid parturients who were admitted with spontaneous labor were enrolled after taking informed consent and screened for eligibility criteria. A convenient sampling technique was used for enrollment. Primigravid parturients aged between 18 and 35 years at term gestation (>37 weeks and <42 weeks) with a single fetus in a cephalic presentation in spontaneous labor were included in the study. Exclusion criteria were the presence of medical complications during pregnancy (diabetes, heart disease, preeclampsia, eclampsia, etc.), malpresentation (face, breech, transverse lie), cephalopelvic disproportion, intrauterine death, and/or diagnosed fetal anomalies. Any contraindications for epidural analgesia such as coagulopathy and neurological disorders were also excluded.

Thirty-five primigravid parturients who fulfilled the eligibility criteria and were willing to receive labor epidural analgesia were allocated to a study group (group S). Thirty-five primigravid parturients who fulfilled the eligibility criteria but refused to receive any analgesia in labor were assigned to the control group (group C). Pregnant women who were booked in our institution were counseled regarding options of epidural analgesia for pain relief during labor.

In the study group (group S, n=35) epidural analgesia was administered to parturients in active labor (once the cervical dilatation reached 4 cm). Preloading was done with Ringer’s lactate 15 ml/kg body weight. An epidural catheter was placed in an upright position in the operation theatre. A multipara monitor was attached to the parturient and baseline parameters were recorded. Taking aseptic precautions L3-4 or L4-5 intervertebral space was identified and 3 ml of 2% lignocaine hydrochloride was locally infiltrated. With 18-gauge (G) Tuohy needle epidural catheter was placed in the epidural space. A bolus dose of 6 ml of 0.1% bupivacaine with 2 mcg/ml fentanyl was given followed by a continuous infusion of the same regimen at the rate of 4 ml/h. Maternal vitals, urine output, and oxygen saturation was recorded throughout labor. An anesthesiologist was present with the parturients of the study group throughout the labor. However, the control group (group C, n=35) comprised primigravid parturients who were not willing to receive epidural analgesia or refused any analgesia.

A similar management protocol was used in both groups. The progress of labor was monitored by maintaining a standard World Health Organization (WHO) partograph. Labor pain intensity was measured using the Visual Analogue Scale (VAS). The VAS is a simple method with high validity and reliability which is frequently employed in patient satisfaction surveys [9]. The ends are defined as the extreme limits of a parameter to be measured (symptom, pain, health). Pain intensity was measured using a scale having pictures of faces depicting the worst type of pain with a score of 10 on one end and a smiling face on another end with a score of 0. Parturients were properly explained about the use of VAS to measure labor pain intensity and were asked to mark on the scale at intervals of 0, 5, 10, 20, 30, 45, 60, 90, 120, 150, 180 min. Zero min in group S corresponded to the time of administration of epidural analgesia in active labor at ≥ 4 cm and in group C when the parturient went into active labor with ≥ 4 cm cervical dilatation. Fetal monitoring was done by continuous intrapartum cardiotocography. Oxytocin infusion was started in case of insufficient uterine contraction. The duration of the active phase of the first stage of labor was calculated from the time of 4 cm dilatation to full dilatation of cervix. The prolonged active phase was defined as a duration of more than 6 h. The duration of the second stage was calculated from full dilatation of cervix to expulsion of baby from birth passage. The prolonged second stage was defined as a duration of more than 2 h. The consultants took the decision to perform a lower segment cesarean section or delivery by instruments (forceps or ventouse). Neonatal outcome was assessed by noting Apgar score at 1 min and 5 min. In all cases, active management of the third stage of labor was done to prevent postpartum hemorrhage (PPH). Follow-up of the mothers and babies was done till discharge from the hospital. Maternal satisfaction in terms of pain relief was assessed as excellent, good, fair, or poor based on subjective sensation within 24 h of delivery.

The primary outcome measure was the alleviation of labor pain measured by the VAS score. Secondary outcome measures were duration of an active phase of the first stage of labor and second stage of labor, mode of delivery, and Apgar score of neonates at 1 min and 5 min.

The sample size was calculated by measuring the difference between the two means. The mean difference was 2.8, with standard deviations of 6.2 and 5.5 in groups S and C, respectively. The confidence interval was 95%, while the expected proportion and precision were 0.07% and 6%, respectively. As a result, the required sample size for each group was computed to be 34 and for convenience, we have taken 35 in each group.

Statistical analysis

Predesigned patient proforma was used to collect data. Different statistical aggregates like mean (average), and standard deviation were used to analyze numerical parameters. Absolute numbers and percentages were used to analyze categorical variables. Statistical analysis was conducted using an unpaired student t-test for parametric variables and a chi-square test for categorical variables. The difference between various parameters among different groups was considered significant if the p-value was < 0.05.

## Results

Seventy primigravid parturients were enrolled for the study as per eligibility criteria. Thirty-five parturients in active labor who received epidural analgesia formed the study group (group S) and 35 cases in active labor not receiving epidural analgesia served as a control group (group C).

Table 1 shows the maternal characteristics of the two groups. The mean age was 21.83±2.61 years in group S and 21.54±4.06 years in group C, mean gestational age was 39.07±1.45 weeks in group S and was 40.07±1.65 weeks in group C, which were statistically not significant. Booking status showed significant difference (71.4% in group S and 40% in group C, p=0.01).

**Table 1:** Demographic profile of primigravid parturients Data are presented as mean±standard deviation (SD) and number (%), group S - study group and group C - control group. *Two-sample independent t-test. **Chi-square test.

Characteristics		Group S (n=35)	Group C (n=35)	p-Value
Age (year)		21.83 ± 2.61	21.54 ± 4.06	0.72*
Gestational age (weeks)		39.07 ± 1.45	40.07 ± 1.65	0.82*
BMI (kg/sq.m)		26.22±3.68	28.57±3.33	0.007*
Booking status	Booked	25 (71.4%)	14 (40%)	0.01**
	Unbooked	10 (28.6%)	21 (60%)	

**Figure 1:**
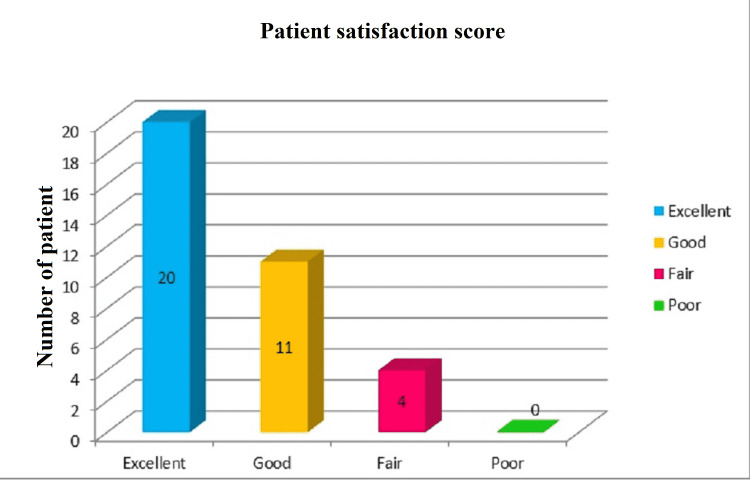
Parturient satisfaction score in study (epidural analgesia) group

Table 2 shows a comparison of VAS scores ranging from 0 to 10 where 0 means “no pain” and 10 means “worst possible, unbearable, excruciating pain” in two groups. The mean VAS score at 0 min in group S (n=35) and group C (n=33) was 7.94 and 7.30, respectively. Two parturients in control group underwent cesarean section for non-reassuring fetal heart rate with meconium-stained liquor in an early active phase, hence were excluded from the assessment of pain score. The mean VAS score quickly dropped to 3.86 in 20 min in group S, while it remained at 7.30 in group C. At 3 h the mean VAS score dropped down to 1.03 in group S, while it increased to 8.39 in the parturients of group C. The difference was statistically significant (p<0.001) at all measured point of time. Thus, the mean VAS score in the study group showed a decreasing trend at all measured points of time indicating a good response of the parturients to epidural analgesia with effective pain control. Figure 1 shows that 31 out of 35 (88.6%) parturients in the study group had high satisfaction scores as excellent and good pain relief based on subjective sensation.

**Table 2:** VAS score of primigravid parturients in two groups Data are presented as mean±SD. *Two-sample independent t-test. VAS: Visual Analogue Scale

Time (min)	Group S (n=35)	Group C (n=33)	p-Value*
0 min	7.94±0.80	7.30 ± 0.85	0.002
20 min	3.86 ±1.03	7.30 ± 0.85	<0.001
30 min	2.31±1.30	7.39 ± 0.79	<0.001
60 min	1.94±1.19	7.52 ± 0.91	<0.001
90 min	1.51±1.01	7.73 ± 0.72	<0.001
120 min	1.26±0.85	7.91 ± 0.72	<0.001
150 min	1.00±0.91	8.27 ± 0.67	<0.001
180 min	1.03±1.07	8.39 ± 0.61	<0.001

Table 3 shows that six (17.1 %) parturients in group S had a prolonged active phase of the first stage of labor (>6 h), prolongation was between 6-12 h. Two (5.7%) parturients in the control group had a prolonged active phase of >6 h, prolongation was between 6 and 9 h. This difference was not significant (p=0.259). The second stage of labor was prolonged >2 h in six (18.2%) parturients group S with variation between 2 h 10 min to 2 h 55 min. No prolongation of the second stage of labor was observed in group C. This difference in duration of the second stage in the two groups was statistically significant (p<0.05).

**Table 3:** Labor/delivery characteristics in two groups Data are presented in number (%), group S - study group and group C - control group. *Fisher's exact test.

Labor/delivery characteristics		Group S	Group C	p-Value
Duration of active phase of first stage (n=35)	≤ 6 h	29 (82.9%)	33 (94.3%)	0.259*
	> 6 h	6 (17.1%)	2 (5.7%)	
Duration of second stage of labor (n=33)	≤ 2 h	27(81.8%)	33(100%)	0.024*
	> 2h	6 (18.2%)	0 (0%)	
Mode of delivery (n=35)	Vaginal delivery	31 (88.6%)	33 (94.3%)	0.550*
	Instrumental delivery	2(5.7%)	0 (0%)	
	Cesarean section	2(5.7%)	2(5.7%)	

Table 3 depicts a comparison of the mode of delivery in two groups. Thirty-one (88.6%) parturients in group S were delivered vaginally, two (5.7%) parturients had a forceps-assisted vaginal delivery, and two (5.7%) parturients underwent cesarean section due to obstetric indications (fetal distress with non-reassuring fetal heart rate in the second stage of labor). Thirty-three (94.3%) parturients in group C delivered vaginally and two (5.7%) parturients underwent cesarean section for non-reassuring fetal heart rate in the first stage of labor. Thus, difference between mode of delivery in group S and group C was not significant (p=0.550).

As shown in Table 4, the mean Apgar score at 1 min was 6.94 and 8.14 in group S and group C, respectively, which was statistically significant. Apgar score improved in the study group at 5 min. Hence, the difference between the 5-minute Apgar score in both groups was not significant (p>0.05).

**Table 4:** Apgar score of neonate Data are presented as mean±SD. *Independent sample t-test.

Apgar score	Group S (n=35)	Group C (n=35)	p-Value*
Apgar score at 1 min	6.94±0.34	8.14±0.69	<0.0001
Apgar score at 5 min	9.00± 0.0	8.94±0.23	0.132

Table 5 represents that the majority (77.1%; 27/35) of the parturients in group S did not have any complications during the intrapartum and postpartum periods. Further, 45.7% of parturients in the control group had nausea and 11.5% (4/35) had giddiness and vomiting mainly due to labor pain. In the study group, 8.6% of parturients had nausea and none of them had vomiting and giddiness. This could be mainly due to significant pain relief provided by epidural analgesia. Two parturients in the study group complained of backache in the postpartum period which was relieved on taking analgesics. This complaint had no relation to the difficult epidural procedure. 

**Table 5:** Comparison of maternal complications between the two groups Data are presented in numbers (%). PPH: postpartum hemorrhage

Adverse effects	Group S (n=35), number (%)	Group C (n=35), number (%)
Nausea	3 (8.6%)	16 (45.7%)
Giddiness	0 (0%)	4 (11.5%)
Vomiting	0 (0%)	4 (11.5%)
Hypotension (transient)	0 (0%)	0 (0%)
Leg weakness	1 (2.9%)	0 (0%)
backache	2 (5.6%)	0 (0%)
Atonic PPH	1 (2.9%)	2 (5.6%)
Retained placenta	0 (0%)	0 (0%)
Dislodgement of epidural catheter	1 (2.9%)	0 (0%)
Uneventful	27 (77.1%)	9 (25.7%)

## Discussion

In the present study, the demographic variables like mean maternal age and gestational age were comparable in the two groups, similar to other studies [10,11]. The booking status in the two groups showed significant differences in contrast to the study by Deshmukh et al. [11]. Booked parturients were counseled about painless labor using epidural analgesia during antenatal visits. Probably, this antenatal education is reflected in increased willingness to receive epidural analgesia. Unbooked parturients in group C were not willing for epidural analgesia probably due to a lack of antenatal education about painless labor. A study by Alakeely et al emphasized the need for education on epidural analgesia in increasing woman’s requests for epidural analgesia [12]. Our study also emphasizes the importance of antenatal counseling in increasing parturient requests for epidural analgesia.

In this prospective non-randomized study, we found a significant reduction in VAS score at all measured points of time in the epidural group versus the control group. The rate of alleviation of pain was also quick, with significantly less pain score achieved in 20 min after administration of epidural analgesia. Our study demonstrated higher satisfaction with pain relief during labor with 88.6% of parturients in the epidural group reporting it to be excellent or good. The finding of our study is supported by other studies, suggesting that epidural analgesia is the gold standard resulting in adequate pain relief and increased patient satisfaction [11,13-15]. Anim-Somuah et al. in their Cochrane review concluded that women receiving epidural analgesia had lesser pain scores with higher satisfaction rates than women receiving opioids or with placebo or no treatment [16].

In our study, the duration of the active phase of the first stage of labor was comparable in group S and group C. This finding is similar to the study by Deshmukh et al. [11]. In contrast, a study showed an increase in the duration of the first stage in nulliparous women by 30 min [17], supported by other studies [14,18]. In contrast, some studies have demonstrated shortening of the first stage of labor with epidural use [10,19]. This is due to better analgesia with the epidural and hence decreased effect of catecholamines to inhibit uterine contractions resulting in faster dilatation of the cervix.

Our study demonstrated prolongation of the second stage (18.2% in group S versus no prolongation in group C), maximum prolongation was of 55 min. Another study has also shown second-stage labor prolongation with the use of epidural analgesia [10]. A study by Naito et al. demonstrated increased duration of both the first and second stages of labor with epidural analgesia [20]. Cochrane review by Anim-Somuah et al. demonstrated longer first and second stages of labor with increased chances of oxytocin augmentation in an epidural group than in the opioid group [16]. However, Deshmukh et al. reported a similar duration of the second stage of the labor in primigravid receiving epidural analgesia and those without any analgesia [11].

In the present study, there was no statistically significant difference in the mode of delivery (cesarean section, vaginal delivery rate, instrument-assisted vaginal birth) between the two groups. This finding is similar to other studies [10,11]. Contrary to this, another study showed that epidural analgesia increases the rate of instrumental vaginal delivery but did not increase cesarean rates [21]. Naito et al. also reported that the cesarean section rate was not statistically different in the epidural group versus the no analgesia group [20]. A study by Hincz et al. found epidural analgesia to be an independent risk factor for instrumental delivery in multiparous women, but no effect on cesarean section rate, in nulliparous or multiparous women [22]. Anim-Somuah et al. in their Cochrane review found a higher rate of instrument vaginal delivery in the epidural group. However, in subgroup analysis after excluding studies before 2005, there was no difference in instrumental delivery and cesarean delivery rate, probably due to the use of lower concentrations of local anesthetics and new techniques of patient-controlled epidural analgesia [16].

In the present study, 1 min Apgar score was found to be lower in the study group but the Apgar at 5 min in the two groups did not show any significant difference similar to previous studies [11,20,22]. Cochrane review by Anim-Somuah et al. also found no differences between groups in neonatal outcomes in terms of Apgar score at 5 min [16].

In the present study, 77.1 % of the parturients group did not have any adverse effects due to epidural analgesia. There was no effect on maternal vitals such as heart rate, blood pressure, and oxygen saturation. The most common side effect in the epidural group was nausea in three cases (8.6%) followed by back pain in two cases (5.6%) and leg weakness in one case (2.9%). No side effects were serious and were managed symptomatically.

The major strength of our study is the inclusion of primigravid parturients less than 35 years at term gestation to account for confounding factors like parity, advanced age, and gestational age in the success of labor. Our study adds to the literature that epidural analgesia provides effective pain relief in labor. Parturients demonstrated improved satisfaction with associated neonatal safety.

The limitation of this study was relatively small sample size. We did not know the level of anxiety of women, the existence of tocophobia, and the level of their psychosomatic preparation for childbirth, especially in unbooked parturients. Another limitation was that perhaps parturients should have been surveyed regarding their future intentions of childbirth showing impact of epidural analgesia on future childbirth. Due to a lack of clear guidelines, which may be related to expertise, cost, and availability of labor epidural analgesia, very few public health facilities in India provide labor analgesia programs. There is a need for larger studies to provide clear guidelines for the use of epidural analgesia for painless labor.

## Conclusions

The present study concluded that epidural analgesia provided excellent and quick pain relief in all primigravid parturients who opted for it. Improved parturients satisfaction with associated neonatal safety provides a positive birth experience. The rates of caesarean section and instrumental delivery were not raised. There was slight prolongation of the duration of second stage of labor but no effect on duration of the active phase of first stage of labor. No major complications were noted in mother. The benefits of painless labor outweighed the side effects.

Epidural analgesia can be implemented as a safe and effective method of pain relief during labor in facilities where expertise for epidural analgesia is available. 
